# Harnessing social media in mental health practice in Kenya: a community case study report

**DOI:** 10.11604/pamj.2021.39.58.27643

**Published:** 2021-05-20

**Authors:** Linnet Ongeri, Gathoni Mbugua, Frank Njenga, Anna Nguithi, Jacqueline Anundo, Maryann Mugane, Zawadi Kimari, Loice Wanjiru Cushny Kaigwa, Lukoye Atwoli

**Affiliations:** 1 Kenya Medical Research Institute, Centre for Clinical Research, Nairobi, Kenya,; 2 Chiromo Medical Centre, Nairobi, Kenya,; 3 Brain and Mind Institute and Medical College, East Africa, Aga Khan University, Nairobi, Kenya

**Keywords:** Social media, mental health awareness, mental health promotion, LMIC, Kenya

## Abstract

The use of social media to increase awareness on mental health is rapidly gaining momentum globally. However, despite evidence of a growing trend in social media use in sub Saharan Africa, little has been reported on tapping the potential of social media within a mental health practice to not only increase awareness but also facilitate linkage to specialist care. We describe one such mental health practice and its process of integration of the different social media platforms to promote mental health and increase linkage to specialist care. We further highlight the challenges and practical implication of social media use in the Kenyan setting. We conclude by advocating for this integration to raise awareness and also encourage peer support for persons with mental health problems and recommend research that measures the impact of such interventions in sub-Saharan Africa.

## Introduction

The impact of social media on mental health has remained controversial. While a number of studies have linked social media to depression and sleep disorders [1] others have gone to argue that indeed social media forms a useful platform for increasing mental health awareness and improving social connectedness [2,3]. Current statistics reveal a growing trend in social media use following increased access to cheaper smart phone devices and Wi-Fi coverage [4]. In January 2020, the average global social media penetrance stood at 49% with an estimated 3 billion social media users [5]. This translates to a 1 in 3 persons use of social media in the world. Even though the sub-Saharan Africa region has the lowest social media use ratings, the continent registered a 12% (+25 million persons) annual growth in the year 2020 [6] emphasizing the fact that trends in social media use are rising even in Africa. Despite the growing concern on the negative mental health effects outlined in recent research it remains unarguable that social media use has pervasively become a way of life for many and especially so following the COVID-19 pandemic whereby physical distancing is a necessity [7-9]. It is then critical as mental health advocates to identify ways of harnessing the power of social media in promoting mental health specifically within our mental health practices. Little information exists on such approaches in the African setting [10] despite the growing numbers of social media users in the region. Our case study seeks to document one such successful approach that may be adopted by others.

## Case study

**Case study setting and population:** Chiromo Mental Health Hospital (CMHH) formerly Chiromo Lane Medical Centre (CLMC) is the oldest private mental health facility in Kenya. Established in 1996 the facility is located in the capital city of Kenya, Nairobi. Referrals come from an admitting base of 28 consultant psychiatrists and 13 clinical psychologists supported by 10 intern psychologist. The hospital caters to both adults and adolescents as in or outpatients. It currently has two branches: the main branch- CMHH Westlands admits acute patients with a bed capacity of 45 patients and the Bustani Branch located in Lavington, Braeside Gardens admits more stable patients, with a bed capacity of 30 patients. Being one of the few established privately run mental health facilities in Kenya, both branches regularly admit close to full capacity. Table 1 shows patient flow in the Chiromo facility. Chiromo mental health hospital specializes in evaluation and treatment of a wide range of psychiatric disorders including; Attention Deficit Hyperactive Disorder (ADHD), Depression, Post Traumatic Stress Disorder (PTSD), Anxiety, and Substance Use Disorders (SUDs). Those with SUDs are referred to a sister organization; the retreat which has two branches in Limuru and Ngong. Chiromo mental health hospital catchment is large with the patient´s base extending as far out into the East African region.

**Table 1:** the monthly average number of inpatients and outpatients managed in Chiromo facility in 2019

CMMH branches	In-patients	Out-patients
**Westlands branch**	37	98
**Braeside branch**	28	200

**Social media platform:** in October 2018, CMHH founded its digital relations department, focusing on the use of social media to normalize and change the narrative surrounding mental health. This decision was influenced by feedback from CMHH´s patients, psychologists and psychiatrists on the scarcity of credible and accessible information within the community on mental health. Many attributed this to the existing high stigma related to mental illness. Given that most of the clientele was between 18-35 years, it came to our realization that these patients consumed information via non-traditional ways. Social media was therefore viewed as a useful tool for disseminating and demystifying mental illnesses as well as providing linkages to care for those in need.

**Digital media team:** the core digital media team consists of three individuals; a clinical psychologist, a psychiatrist and a digital strategist. The psychologist leads the digital relations department and is responsible for forging partnerships between other organizations and CMHH on online platforms, participating in driving the conversation on mental health on national and international awareness days as well as overseeing content that will go into the various platforms. The content to be shared is determined ahead of time and incorporated into a content calendar which remains flexible and can be altered based on the emerging needs in the population as perceived by the psychiatrist and psychologist. The digital strategist is responsible for developing digital posters, shooting and editing short videos, taking photos and posting on media platforms as advised. The psychiatrist gives feedback on the content posted online and guides the team on professional growth from his wealth of knowledge in mental health. The social media content is primarily driven by the general public. The team receives suggestions on specific topics of interest on the various platforms and holds polls to prioritize on key public health issues to be addressed. Chats and comments are not censored as unbiased opinion from the public often offers an opportunity for engaging more persons and dispelling existing myths. However, messages deemed insulting to a specific individual are deleted. Chiromo mental health hospital social media presence has now been established on various platforms.

**Twitter:** in 2018, the digital media team began the mental health conversation on twitter (@ChiromoMentalHH). Twitter was selected for its extensive reach capabilities as well as the provision to have real time interaction. The psychologist, the digital strategist together with the institutions chairman and senior consultant psychiatrists developed a list of the most common mental illnesses managed at the facility namely; ADHD, depression, bipolar mood disorder, schizophrenia, anxiety disorders as well as substance use disorders. Discussions were held outlining the signs and symptoms, myths surrounding the specific illness, when, where and how to receive help. To date, the topic areas are selected based on current and most pressing issues and suggested topics from the community. Each tweet chat runs for an hour and is led by two psychologist panelists of different gender to allow for gender representation. The panelists are either from CMHH or guest panelists through invitation. Presently, three live tweet programs are ongoing under the twitter handle (@ChiromoMHH). One runs a live tweet every Monday for an hour with the hashtag #MindfulMondays, a second one is a regional tweet chat with panelists from various African countries with the hashtag #Mentalhealth4Africa. The last and most recent runs daily and the focus for this is on COVID impact on mental health. Thus far the twitter following has grown exponentially starting in 2018 with 218 followers to current standing at 2245. The tweet chats hold an average of 40,000 impressions (number of times content was viewed) and 80,000 tweet reach per week. Visits per tweet chat stand at 984. For the conversation to reach as many people as possible, a number of direct messages are sent to various mental health practitioners and organizations online, highlighting the topic, their role as a panelist, the time and duration as well the fact that their participation would be on a volunteer basis. Those who respond, are looped in as panelists, and are requested to share their bio and photo. Posters are then created by the digital strategist to publicize the chat and are shared with the panelists who can also use them in their own social media platforms to inform their followers. The posters are also circulated to the various heads of departments in CMHH to share with their team members, who can also share with others. The live tweet chats are not limited to the panelists only, instead panelists help direct the conversation and address questions brought up by the attendees for the day. While the live chat would end at a specified time, the lead psychologists and digital strategists look through the platform throughout the day to answer any questions that come up from those who may have joined the conversation later. One limitation of twitter is existing word limit hence, for longer texts, Facebook is preferred.

**Facebook:** Facebook (Chiromo Mental Health Hospital) has over 32,000 followers with engagement and reach of 386 to 3,955 on weekly posts mainly by female followers (68%). Facebook´s content is both pictorial and text and takes the cue from the twitter conversation held on #MindfulMondays. Within the week´s duration, pictures, posters and relevant information is posted on this platform daily to keep the conversation going and in line with that week´s or month´s theme. Any questions raised on the platform are answered both in the textbox and through direct messaging. This platform has the capacity to hold long videos. Chiromo Hospital Live is a segment created within the Facebook page, to post videos done by in house hospital psychologists and psychiatrists, where discussions on mental health issues e.g. rising cases of suicide are discussed, recorded and posted. Recently started is the men´s mental health live video conversation. This is led by the institution´s male psychologists. The traffic on the Facebook platform also has a wider reach extending as follows; Ethiopia 53%, Tanzania 27%, Somalia 16%, Kenya 15% and from Uganda 11%. For this reason, Facebook platform is often used to boost posts and send videos.

**Instagram:** Instagram (@chiromoMentalHosp) has 626 followers of whom 54% are female. Due to the visual focus it is the least active platform. Despite this it still boasts a wider reach extending as far out at the US. Designed for a younger age group (18-24 yrs), this platform focuses on pictures, short videos, and Gifs uploads. Posters on the theme of the month are uploaded on the platform. These posters carry both graphical youth friendly images and simple wording that take the format of tips for example; 10 tips to cope with anxiety. Insta stories is another feature on Instagram that has proven useful in receiving feedback from our followers through set up polls. These statuses are short lived and only last for 24 hours before self-deleting hence necessitating constant checking and responding to queries. Overall, Instagram remains a useful platform for raising reflective questions, sharing pictures of events held or sponsored by the hospital as well as conducting polls on what content people would like to see in relation to mental health.

**Figure 1:**
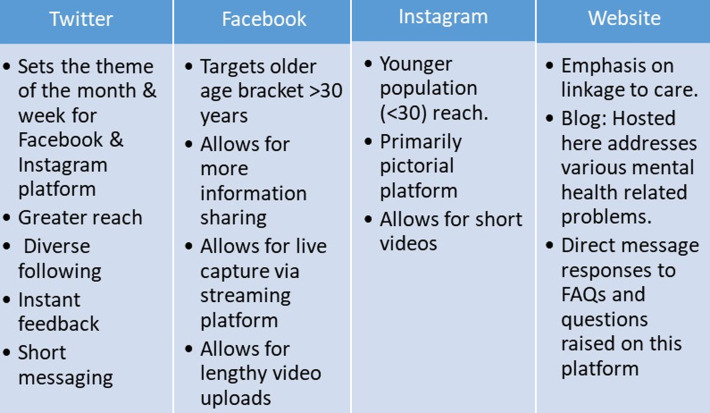
summary of the various social media platforms used in CMHH

**Website:** traffic from other social media platforms is redirected to the facility website where users are able to access all information regarding the institution. Clients are engaged through already set FAQs and queries are answered in real time. The website receives an average of 1100 visitors with an average of 82.2% new visitors and 17.8% returnees per month. Age group of most frequent visits are between 25-34 years. The top three countries to visit the website are 82.6% Kenya, 6.5% US and 1.8% UK with a gender representation of 61.9% female and 38.1% male. The website additionally hosts a monthly blog regarding mental health issues. Figure 1 presents a summary of the pros and cons of the various social media platforms used in CMHH.

**Practical implications:** although, the use of social media platforms to increase awareness on mental health problems is both convenient and cost effective, curating messages and engaging users can be time consuming and a high level of consistency is required to ensure sustainability. Hence, a team approach is necessary in running a successful awareness program. Furthermore, the digital media space is very dynamic both in information and in its technical usage, hence it is pertinent that the team members remain updated on current topical issues in the country relevant to the different targeted age groups as well as maintain an element of tech savviness to ensure relevance. Overall, the use of social media in our setting has played a key role in breaking the barriers of information sharing on mental health matters, allowing for greater engagement with care and services and reducing existing stigma that is related to lack of knowledge. In addition, social media use has been resourceful in offering a sense of community or support system to persons affected both directly and indirectly by mental health challenges.

**Lessons learned:** messaging is very key and every detail needs to be analyzed and reanalyzed and then packaged into a format that is appealing for the audience in each of the various platforms. Overall, simple messaging, free from clinical jargon works best for psychoeducation. Furthermore, inclusion of persons from diverse demographic backgrounds including; gender, age and occupation in the planning and disseminating of content is crucial to improve on content authenticity, relevance and reach. Lastly, virtual networking by identifying persons pursuing similar goals and supporting them too is key in ensuring growth and sustenance of one´s social media presence.

## Conclusion

The use of social media platforms to combat stigma and increase awareness of mental health problems though feasible does harbor some conceptual and methodological constraints. We have earlier alluded to evidence provided by a number of studies linking prolonged use of social media to symptoms of depression, anxiety and addiction [11], hence, it may appear counterintuitive to use the same platform to address these same problems. However, partly due to the perceived anonymity and convenience of social media use, research has shown a high number of persons with mental health problems are increasingly turning to social media to share experiences and to seek advice [12,13]. Thus, despite the potential drawbacks, the role of structured social media usage by mental health providers to facilitate help seeking behavior and promote peer support cannot be overstated. Our experience demonstrates that we can harness this platform to increase awareness on mental health, counter misinformation and link persons to care. Future research examining the effectiveness of mental health promotion through social media platforms in increasing linkage to care is needed to further understand its role in reducing the mental health treatment gap in sub-Saharan Africa.
